# Thermoelectric Properties of Cu_2_Se Synthesized by Hydrothermal Method and Densified by SPS Technique

**DOI:** 10.3390/ma14133650

**Published:** 2021-06-30

**Authors:** Paweł Nieroda, Anna Kusior, Juliusz Leszczyński, Paweł Rutkowski, Andrzej Koleżyński

**Affiliations:** 1 Department of Inorganic Chemistry, Faculty of Materials Science and Ceramics, AGH University of Science and Technology, al. A. Mickiewicza 30, 30-059 Krakow, Poland; akusior@agh.edu.pl (A.K.); jleszczy@agh.edu.pl (J.L.); 2 Department of Ceramics and Refractories, Faculty of Materials Science and Ceramics, AGH University of Science and Technology, al. A. Mickiewicza 30, 30-059 Krakow, Poland; pawelr@agh.edu.pl; 3 Department of Silicate Chemistry and Macromolecular Compounds, Faculty of Materials Science and Ceramics, AGH University of Science and Technology, al. A. Mickiewicza 30, 30-059 Krakow, Poland; andrzej.kolezynski@agh.edu.pl

**Keywords:** copper (I) selenide, thermoelectric materials, spark plasma sintering, thermoelectric properties

## Abstract

The aim of the work was to obtain copper (I) selenide Cu_2_Se material with excellent thermoelectric properties, synthesized using the hydrothermal method and densified by the spark plasma sintering (SPS) method. Chemical and phase composition studies were carried out by X-ray diffraction (XRD), scanning electron microscopy (SEM), and transmission electron microscopy (TEM) methods. Measurements of thermoelectric transport properties, i.e., electrical conductivity, the Seebeck coefficient, and thermal conductivity in the temperature range from 300 to 965 K were carried out. Based on these results, the temperature dependence of the thermoelectric figure of merit *ZT* as a function of temperature was determined. The obtained, very high *ZT* parameter (*ZT*~1.75, *T* = 965 K) is one of the highest obtained so far for undoped Cu_2_Se.

## 1. Introduction

Cu_2_Se is one of the most intensively studied thermoelectric materials in recent years, suitable for the construction of thermoelectric generators that work in a medium temperature range (600–950 K) [1]. The basic parameter describing the thermoelectric properties of materials is the thermoelectric figure of merit *ZT*, *ZT* = α^2^σλ^−1^T, where α is the Seebeck coefficient, σ is the electrical conductivity, λ is the thermal conductivity, and *T* is the temperature. A value of the *ZT* parameter is directly related to the efficiency of thermoelectric devices such as thermoelectric generators and thermoelectric heat pumps, which increases with increasing *ZT*. In practice, it is assumed that materials that are interesting in terms of thermoelectric applications should have a *ZT* parameter that is equal to at least one. Materials based on Cu_2_Se have very high values of the *ZT* parameter [2,3], comparable with the values obtained for the best, far more thoroughly tested materials from other groups (like skutterudites, clathrates etc. [4]), e.g., *ZT* = 2.0 for Cu_2_Se/0.05 wt.% SiC, *T* = 873 K [5], *ZT* = 1.98 for Cu_2_Se + 0.8 wt.% CDs (carbon nanodots) *T* = 973 K [6], *ZT* = 2.6 for Cu_2_Se + CuInSe_2_ inclusions, *T* ≤ 850 K [7], *ZT* = 2.14 for Cu_1.98_Li_0.02_Se, *T* = 973 K [8], and *ZT* = 2.62 for Al doped Cu_2_Se, *T* = 1029 K [9]. The excellent thermoelectric properties of materials based on Cu_2_Se are the result of their specific properties, describable using the phonon-liquid electron-crystal PLEC concept [10,11], which is an extension of the phonon-glass electron-crystal PGEC concept [12]. According to PLEC, in Cu_2_Se having a regular calcium antifluoride crystal structure, Se atoms occupy 4a Wyckoff’s position and form an anionic framework, ensuring free movement of electron carriers through crystal lattice nodes, while very mobile copper ions (with diffusivity at 10^−5^ cm^2^s^−1^ *T* = 430 K for Cu_2_Se [13]) forming cationic sublattice can easily “jump” at short time intervals between equivalent positions (8c and 32f) in crystal lattice [10]. This leads to a strong phonon scattering and results in a very low thermal conductivity in these materials (comparable to the thermal conductivity of glasses) and, additionally, increased electrical conductivity [4,10]. Migration of Cu^+^ ions in Cu_2_Se-based materials is responsible not only for very good thermoelectric transport properties, but unfortunately also for the lack of stability of these materials under current load, which was a subject of the works [14,15,16]. The problem of chemical stability (independently of the method of synthesis and densification) concerns thermoelectric materials from various groups. Therefore, intensive research is carried out to increase the stability of these types of materials, e.g., by doping, changing the microstructure, or creating the composite materials, as seen in [17,18]. Cu_2_Se is mainly synthesized in polycrystalline form and then sintered by various techniques. In some studies [10,19,20], high-temperature synthesis in quartz ampoules was used and the sintering of powders was performed using spark plasma sintering SPS [10,19] or the conventional sintering CS [20] method. In other studies [5,21,22,23], a synthesis was carried out using the mechanical alloying MA technique, and sintering by hot pressing HP [21,22] or SPS [5,22,23] method. A very quick, one-step method for the preparation of Cu_2_Se using arc-melting was proposed by Butt et al. [24].

In this work, an alternative method of obtaining Cu_2_Se powders with the hydrothermal method and subsequent densification using the SPS technique is proposed, allowing for the obtaining of materials with excellent thermoelectric properties comparable and often better than those obtained with other techniques in the entire studied temperature range.

## 2. Materials and Methods

Cu_2_Se nanopowders were synthesized by means of the hydrothermal technique. Firstly, 0.002 mol of CuSO_4_·5H_2_O (Chempur, Piekary Śląskie, Poland) and 0.01 mol of selenium oxide (Acros, Geel, Belgium) were dissolved in distilled water (160 mL). After 10 min stirring, prepared solution was sonicated for 15 min. Afterward, the 20 mL of the hydrazine hydrate (81%, Sigma Aldrich, St. Louis, MO, USA) was dropwise added to the mixture and transferred into Teflon-line autoclave, where it was kept for 24 h at 200 °C. The black product was collected by centrifugation, washed with ethanol solution (50% v/50% v, H_2_O/EtOH), and dried at 60 °C in a vacuum.

The powders after synthesis were densified by the SPS (Spark Plasma Sintering) technique in graphite dies at various temperatures (*T* = 823 K or 923 K or 993 K, *p* = 50 MPa, *t* = 1 min, vacuum *p* = 10^−3^ mbar, heating rate *v* = 100 K·min^−1^). The densities of the samples measured by the hydrostatic method were equal to 4.84 g·cm^−3^ (*T* = 823 K), 5.58 g·cm^−3^ (*T* = 923 K) and 6.02 g·cm^−3^ (*T* = 993 K), respectively. The phase composition analysis was performed using the X-ray powder diffraction method (X-ray Diffractometer Panalytical Empyrean (Malvern, Worcestershire, United Kingdom), CuKα, λ = 1.5418 Å). The morphology and chemical composition of the as-synthesized nanopowders were characterized by a Nova NanoSEM 200 (FEI COMPANY, Hillsboro, Oregon, United States)) scanning electron microscope (SEM) and a Tecnai TF 20 X-TWIN (FEI) high-resolution transmission electron microscope (TEM) with an integrated energy dispersive spectrometer (EDX). Gatan DigitalMicrograph^®^ Software (GMS 3.4.3, Gatan, Inc., Pleasanton, CA, USA) was applied to determine d-spacing values and distinguish lattice fringes of different phases. Electrical conductivity and the Seebeck coefficient were determined in the temperature range 300–965 K using a homemade apparatus. Electrical conductivity was measured using a four-probe method with variable DC polarization. Seebeck coefficient measurements were carried out using a small (Δ*T* < 4 K), forced temperature gradient across the specimen. Thermal conductivity was studied by the laser flash method LFA (Laser Flash Apparatus LFA 427, Netzsch, Selb, Germany) in the temperature range 300–965 K. The heat capacity of the material was determined in thermal diffusivity measurements, and it was consistent with the heat capacity reported by Liu et al. [10].

## 3. Results and Discussion

### 3.1. Structural and Microstructural Analysis

**Figure 1:**
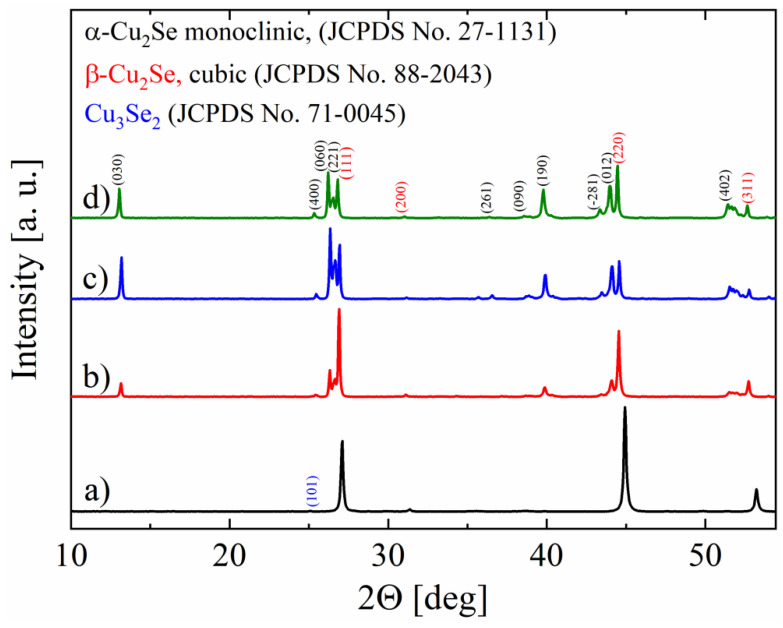
X-ray diffraction patterns for Cu_2_Se samples after synthesis (**a**) and after sintering by the SPS method at 550 °C (**b**), 650 °C (**c**), 720 °C (**d**).

The material obtained after hydrothermal synthesis consists mostly of cubic Cu_2_Se. The X-ray diffraction pattern (Figure 1) shows small amounts of impurities in a form of Cu_3_Se_2_ (JCPDS No. 71-0045) and CuO (JCPDS No. 89-5898). After sintering, structural changes occur in the material and with increasing sintering temperature, the share of the α-Cu_2_S monoclinic phase increases at the expense of the β-Cu_2_S cubic phase. The presence of the sole cubic phase in the powder after synthesis distinguishes the hydrothermally obtained material from the copper selenide obtained by direct high-temperature synthesis, where a mixture of α-Cu_2_S and β-Cu_2_S, with the predominance of the monoclinic phase, is always obtained. However, as can be observed during sintering, this state is metastable.

**Figure 2:**
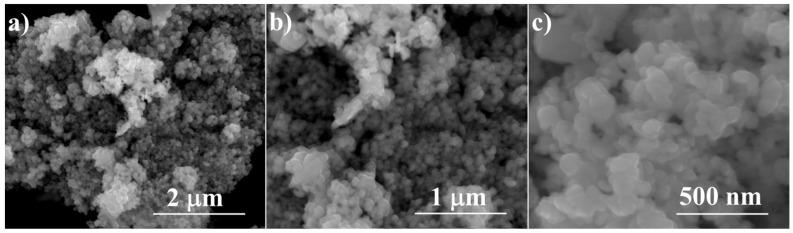
SEM photographs of Cu_2_Se powders after synthesis at magnification: (**a**) 50.000× (**b**) 100.000× and (**c**) 200.000×.

**Figure 3:**
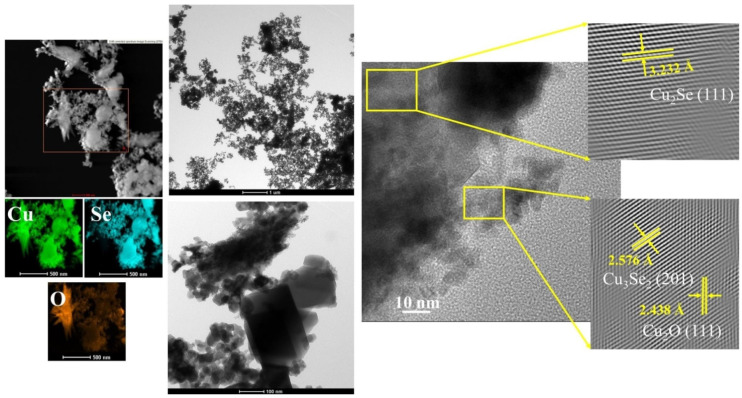
TEM results of Cu_2_Se samples.

The microstructural analysis was performed using a SEM and TEM (Figure 2 and Figure 3). SEM images indicate that the nanopowder consists of very small grains, which cannot be fully observed using this technique. Therefore, high-resolution transmission electron microscopy (HR-TEM) imaging was employed.

Figure 3 presents more detailed microstructural studies. It is shown that the powder consists of variously shaped and sized grains. Observed in larger grains, lattice fringes of about 3.232 Å can be assigned to the (111) crystal facet of Cu_2_Se, while differently oriented lattice spacing of about 2.576 Å and 2.438 Å may correspond to smaller Cu_3_Se_2_ (201) and Cu_2_O (111) particles, respectively. XRD measurements proved the presence of Cu_3_Se_2_ intermediate phases. Energy-dispersive X-ray (EDX) mapping reveals that copper and selenium are homogeneously distributed in the powder, while oxygen content is located at a border of larger grains. It can be assumed that the used mole fraction of the components promotes the formation of copper oxide and/or that the material has been oxidized during its preparation.

**Figure 4:**
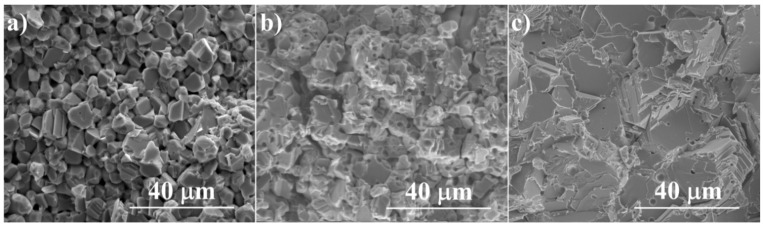
SEM photographs of the surface of cross-section fractures for Cu_2_Se samples sintered at (**a**) 550 °C, (**b**) 650 °C and (**c**) 720 °C.

Figure 4 shows images of cross-section fractures of Cu_2_Se samples sintered at different temperatures. The sample sintered at 550 °C has the smallest grains but also the highest porosity. The grains adhere to each other only partially through the necks formed, while between the grains there are pores that combine to form an open network of channels. The grains from the initial Cu_2_Se nanopowder have grown to a diameter of 4–10 μm. Raising the sintering temperature to 650 °C results in better compaction of the material accompanied by further grain growth. The grains get much closer to each other and their contact area increases. A large number of pores is still visible at the grain boundaries; they are mostly closed, and their volume decreases, which indicate that an intermediate sintering stage has been reached. The temperature increase to 720 °C results in further densification; however, a small amount of copper oxides, most probably in the form of nanocrystallites or thin passivation layers on Cu_2_Se grains, inhibits to some extent the pore elimination process. As can be seen in the microphotograph, a very large growth of grains to diameters in the range of 30–60 μm is undergone, accompanied by a significant disappearance of pores. Few pores are located at grain boundaries, while some of them are located inside grains. One can expect that a higher density of the sintered samples and a smaller number and volume of pores will have a positive effect on electrical conductivity (its increase), without changing the value of the Seebeck coefficient, which depends primarily on carrier concentration.

### 3.2. Thermoelectric Transport Properties

During transport properties measurements of the obtained samples, noticeable changes in all characterized properties were observed between the first and subsequent measurements. Therefore, presented results include the first four or three measurements, which display the evolution of the changes in the properties and their stabilization in subsequent measurements.

**Figure 5:**
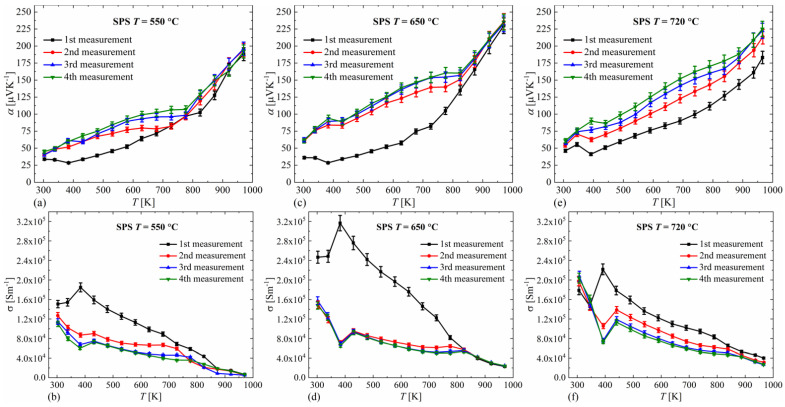
Temperature dependence of the Seebeck coefficient and the electrical conductivity for Cu_2_Se samples sintered at 550 °C (**a**,**b**), 650 °C (**c**,**d**), 720 °C (**e**,**f**) (absolute errors for *α*_r_ = 0.05).

All the samples obtained, for each of the measurements performed over the entire range of temperatures studied, have positive Seebeck coefficient values that increase with temperature, as shown in Figure 5a,c,e). The temperature dependence of the Seebeck coefficient for the first measurements shows lower values than for the second and subsequent measurements. Similar results are presented in [25]. The change in this dependence between the second and third measurements is small, while the third and fourth measurements are practically equal in terms of measurement error. Therefore, in the following discussion, we will refer to the results of measurements #3 and #4. For all samples, inflection points can be observed in temperature dependence α(T), at around 396 K (which corresponds to the phase transition between α- and β-Cu_2_Se) and around 800–850 K. Variations in the monotonicity of the temperature dependence of the Seebeck coefficient near the phase transition temperature are observed in most studies [1,2,3]. The sample sintered at 550 °C has slightly lower Seebeck coefficient values than samples sintered at 650 °C and 720 °C. This may be due to a slightly different ratio of the monoclinic phase to the cubic phase or minor changes in stoichiometry or microstructure. The observed Seebeck coefficient values are similar to those reported for Cu_2_Se densified by the SPS method [20,21,22]. Compared to the results of other authors [26] who obtained Cu_2_Se by the hydrothermal method, the Seebeck coefficient has very similar values in the whole range of measured temperatures.

For all of the samples, significantly higher values of electrical conductivity σ were observed in the first measurement compared to subsequent measurements (Figure 5b,d,f), and like for the Seebeck coefficient measurements, measurements #3 and #4 were virtually identical, and thus will be discussed further. Obtaining reproducible values of the Seebeck coefficient and electrical conductivity for successive measurements shows that Cu_2_Se is a fairly stable material in the temperature range studied. The temperature dependence of the electrical conductivity of all samples is metallic-like, and the conductivity decreases with temperature. This is a typical conductivity relationship observed for materials with a metallic nature of conductivity, where the carriers mobility decreases with increasing temperature. Similar to the Seebeck coefficient measurements, a spike in conductivity can be observed at around 390–400 K, which can be attributed to Cu_2_S phase transition, and an inflection of the curve is also noticeable around 800–850 K. As the sintering temperature increases, an increase in conductivity is observed, which is probably primarily due to a higher material density and grain growth, as an increase in sintering temperature is accompanied by an increase in the Seebeck coefficient, indicating, rather, a decrease in carrier concentration. The similar Seebeck coefficient and electrical conductivity values for the samples sintered at 650 °C and 720 °C indicate similar defect concentrations in both samples. The obtained values of electrical conductivity are similar to the results of other researchers [20,21,22]. The differences we observe with respect to the results of Gao et al. [26] for hydrothermally obtained Cu_2_Se can be easily explained by differences in carrier mobility, as seen by the similar Seebeck coefficient in our samples. Only a few works [23] find dense bulk Cu_2_Se having higher Seebeck coefficients and lower electrical conductivity, which could be attributed to a lower concentration of Cu vacancies. The high Seebeck coefficient values and phase transition temperature close to 396 K show that despite the small amounts of impurities present due to the synthesis method used, the phase composition does not show large Cu deficiency and is close to stoichiometric. The inflections appearing at 800 K on the dependences α(T) and σ(T) may indicate the appearance of an additional carrier scattering mechanism.

**Figure 6:**
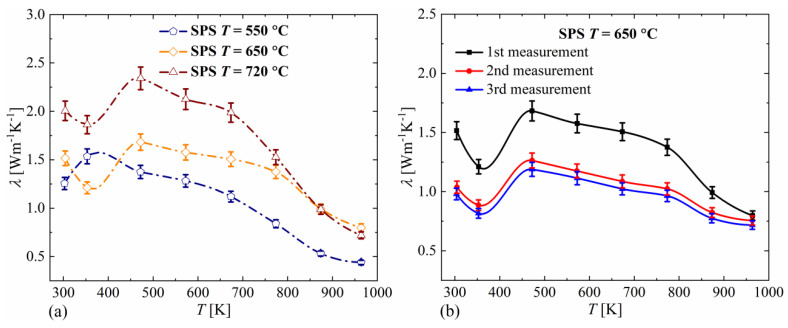
Thermal conductivity for Cu_2_Se samples sintered at different temperatures (**a**) and for different measuring cycles for the sample sintered at 650 °C (**b**) (absolute errors for *α*_r_ = 0.05).

In the case of the thermal conductivity measurements we can observe, similarly to electrical conductivity and the Seebeck coefficient, a significant change in the conductivity values between the first and second measurements as shown for the sample sintered at 650 °C (Figure 6b). The thermal conductivity decreases, which corresponds well with the decrease in electrical conductivity between the first and second measurements. This also shows that an electronic component is primarily responsible for the thermal conductivity of Cu_2_Se. The thermal conductivity results shown in Figure 6a demonstrate that the sintering temperature affects the thermal conductivity of the materials obtained.

Similar to the other transport properties, at a temperature around 400 K, a stepwise decrease in thermal conductivity is observed due to a phase transformation. Above this temperature, the thermal conductivity decreases, which is consistent with observations presented in other works [19,20,21,22]. At 800 K, there is a slight inflection of this relationship and a faster decrease in thermal conductivity, corresponding to the inflection in the electrical conductivity relationship.

**Figure 7:**
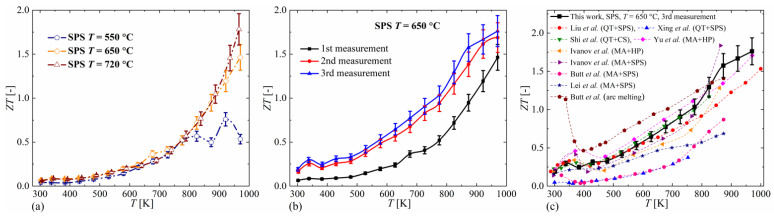
Temperature dependence of *ZT* parameter for Cu_2_Se samples sintered at different temperatures (**a**), for different measuring cycles for the sample sintered at 650 °C (**b**) and for the sample sintered at 650 °C with literature data (**c**) (absolute errors for *α*_r_ = 0.10), MA-mechanical alloying, QT-high temperature synthesis in quartz tube, SPS-spark plasma sintering, HP-hot pressing, CS-conventional sintering.

The thermoelectric efficiency coefficient *ZT* was calculated based on measured transport properties. The obtained *ZT* values, determined using the results of the first measurement of electrical conductivity and the Seebeck coefficient, and the first measurement of thermal conductivity, are presented in Figure 7a. Over a wide temperature range, the *ZT* values do not depend on the sintering temperature. Only at temperatures above 800 K does the sample sintered at 550 °C have significantly worse properties, while the other two samples have *ZT* values close to each other within a measurement error, reaching the maximum value of about 1.75 at 970 K for the sample sintered at 720 °C. For comparison, *ZT* results for the sample sintered at 650 °C calculated for the first, second and third measurements, are also included (Figure 7b). It can be seen that between the first and second measurements the *ZT* value increases, and between the second and third measurements the differences obtained are within an experimental error. Therefore, the *ZT* results presented in Figure 7a should be considered as underestimated. The obtained *ZT* values are among the highest ones presented in the literature for copper selenide (especially for higher temperatures) and are comparable with *ZT* values found for other materials obtained from nanopowders. The properties of Cu_2_Se obtained by direct synthesis, except for arc melting, generally show much worse thermoelectric properties (Figure 7c). Compared with previous studies of Cu_2_Se obtained by the hydrothermal method [26], three times higher *ZT* values were obtained, which is mainly due to the higher electrical conductivity and Seebeck coefficient of the obtained materials. Since the Seebeck coefficient in our samples and those of Gao et al. are similar, it can be assumed that these differences are mainly due to the presence of microstructural defects.

## 4. Conclusions

Cu_2_Se was synthesized by the hydrothermal method and sintered under various conditions using the SPS method. The obtained samples were homogeneous in terms of their chemical and phase composition, as confirmed by XRD and SEM results. Careful TEM testing, however, shows the presence of small amounts of copper oxide phases that can hinder sintering. As the sintering temperature increased, an increase in pellet density and an improvement in thermoelectric properties expressed by the *ZT* parameter were observed, whereby the samples sintered at 650 °C and 720 °C had very similar values of the *ZT* parameter (within the measurement error limits). It was shown that all obtained samples change their transport properties during cyclic measurements and show stability from the third measurement cycle. The applied synthesis method and subsequent SPS sintering allowed for the obtaining of materials with very good transport properties and *ZT* parameter, with the latter values comparable to the best reported for such materials obtained employing other techniques. The obtained results show the high practical potential of the hydrothermal method for the syntheses of thermoelectric materials from the selenide group with very good transport properties.

## Data Availability

Data sharing is not applicable to this article.
